# The Value of ^68^Ga-PSMA PET/CT Following Equivocal ^18^F-NaF PET/CT in Prostate Cancer Patients

**DOI:** 10.3390/diagnostics10060352

**Published:** 2020-05-28

**Authors:** Claus Madsen, Peter Østergren, Christian Haarmark

**Affiliations:** 1 Department of Nuclear Medicine, Copenhagen University Hospital Herlev and Gentofte, DK-2730 Herlev, Denmark; christian.eickhoff.haarmark.nielsen.01@regionh.dk; 2 Department of Urology, Copenhagen University Hospital Herlev and Gentofte, Borgmester Ib Juuls Vej 1, DK-2730 Herlev, Denmark; peter.busch.oestergren@regionh.dk

**Keywords:** PSMA PET/CT, NaF PET/CT, prostate cancer, bone metastases

## Abstract

Background: Inconclusive bone scans are a challenge but there is no consensus about follow-up imaging. We evaluated the use of ^68^gallium-labelled prostate-specific membrane antigen (^68^Ga-PSMA) PET/CT if ^18^F-sodium fluoride (^18^F-NaF) PET/CT was inconclusive. Methods: This retrospective study included patients with no previously known bone metastases who had one or more equivocal bone lesions on ^18^F-NaF PET/CT and underwent additional ^68^Ga-PSMA PET/CT. The bone lesions were deemed as true metastases or not based on follow-up by surveying supplemental imaging modalities and hospital records. A subgroup of patients with “most valid follow-up” was created, which included patients with unmeasurable PSA after prostatectomy or subsequent imaging (additional ^18^F-NaF PET/CT, ^68^Ga-PSMA PET/CT, CT, or MRI). Results: Of the 2918 patients referred for ^18^F-NaF PET/CT from the department of urology in the inclusion period, 51 (1.7%) were inconclusive regarding bone metastases and underwent additional ^68^Ga-PSMA PET/CT. Thirteen of these patients (25%) were ultimately diagnosed with bone metastases. Patient-based sensitivity, specificity, and accuracy of additional ^68^Ga-PSMA PET/CT were 100%, 95%, and 96%, respectively. In patients with “most valid follow-up”, the same parameters were 100%, 93%, and 94%, respectively. Conclusion: ^68^Ga-PSMA PET/CT is an excellent complementary modality in when ^18^F-NaF PET/CT is equivocal.

## 1. Introduction

Prostate cancer (PC) is one of the most frequent cancers in men worldwide. Bone is the most common site of distant metastases [1] and is associated with increased morbidity with skeletal-related events and poor survival [2]. Bone scintigraphy is currently the recommended modality for the diagnosis of bone metastases [3,4]. However, many institutions provide ^18^F-sodium fluoride PET/CT (^18^F-NaF PET/CT) instead of bone scintigraphy. Both bone scintigraphy and ^18^F-NaF PET reflect osteoblastic activity, which is often increased in bone metastases of PC [5,6]. One advantage of ^18^F-NaF PET/CT is that the number of equivocal scans is reduced [7,8]. However, since both malignant and benign lesions often have sclerotic potential, it is inevitable that some ^18^F-NaF PET/CT scans will be inconclusive.

^68^Ga-labelled prostate-specific membrane antigen (^68^Ga-PSMA) binds to the PSMA glycoprotein on the cell surface [9]. The density of the PSMA-glycoprotein is elevated in PC cells, and, in contrast to ^18^F-NaF, ^68^Ga-PSMA directly reflects the malignant cells in the tumor [10]. The use of ^68^Ga-PSMA PET/CT in soft-tissue lesions is well established in primary staging of high-risk patients [11,12,13,14] and in patients with biochemical recurrence [15,16,17,18]. Knowledge concerning ^68^Ga-PSMA PET/CT regarding bone metastases is more limited. However, the potential of this tracer to evaluate bone metastases seems promising [19,20].

As the diagnostic conclusion of bone metastasis has major impacts on treatment and prognostic evaluation in men with PC, inconclusive bone scintigraphy or ^18^F-NaF PET/CT is problematic and confusing, and potentially leading to delayed or even inappropriate treatment. The purpose of this study was to investigate the use of ^68^Ga-PSMA PET/CT as a supplemental imaging method when ^18^F-NaF PET/CT is inconclusive and to determine the anatomic locations of equivocal lesions on ^18^F-NaF PET/CT.

## 2. Materials and Methods

### 2.1. Patients

This retrospective single-center study comprised of ^18^F-NaF PET/CT scans performed from 26-06-2017 to 08-11-2018 on PC patients referred from the department of urology in a large tertiary hospital (Copenhagen University Hospital Herlev and Gentofte, Herlev, Denmark). Included patients had no known bone metastases but an inconclusive ^18^F-NaF PET/CT and were referred to subsequent ^68^Ga-PSMA PET/CT. The project was approved on 11-04-2018 by the local administration. No Ethic Committee approval was necessary.

### 2.2. ^18^F-NaF PET/CT

^18^F-NaF was injected 30 min before image acquisition. The targeted injected dose was 200 MBq. PET/CT was performed with Biograph mCT (Siemens Healthineers, Erlangen, Germany). Images were obtained from the top of the skull to just below the knees. The acquisition time was 1 minute/bed position. The 18F-NaF PET was combined with either “low-dose” (tube current–time product 40 mAs with dose modulation) or “diagnostic” (tube current–time product 180 mAs with dose modulation) CT if evaluation of soft tissue was indicated. If diagnostic CT was performed, iodinated contrast was infused if tolerated. The choice between non-diagnostic and diagnostic CT was left to the discretion of the referring urologist. All scans were reviewed by an experienced nuclear medicine physician. The diagnostic CT scans were reviewed by an experienced radiologist, and the low-dose CT scans were reviewed by a radiologist if requested. Additional details of the examination protocol was described in another study [20].

### 2.3. ^68^Ga-PSMA PET/CT

Approximately 60 min before image acquisition, a bolus of 2 MBq/kg of ^68^Ga-PSMA-11 was injected via a peripheral venous catheter positioned in an antecubital vein. PET/CT was performed with Biograph mCT (Siemens Healthineers). Images were obtained from the top of the skull to just below the knees. The acquisition time was 3 min/bed position. Additional details of the examination protocol was described in another study [20]. All CT scans were “diagnostic”, as dictated by the routine protocol. A CT contrast agent was administered if not contraindicated.

### 2.4. Lesions on ^18^F-NaF PET/CT

All scans were interpreted by nuclear physicians with high experience as part of clinical routine. A lesion was deemed equivocal if the appearance was neither typically benign nor malignant. Decision criteria included intensity of uptake (naïve bone metastases are typically highly fluoride avid), anatomical location of lesion (long distance from prostate interpreted as more likely benign), and bone type (lesion in cancellous bone more likely malignant than in compact bone). Based on the most typical locations of bone metastases [21], the locations of the lesions were subdivided into seven anatomical categories: The pelvis, the lumbar vertebrae, the thoracic vertebrae, the femora, the ribs, other sites, and multiple regions (if lesions were located in 2 or more of the listed regions).

### 2.5. Follow-Up

Equivocal bone lesions were deemed as true metastases or not based on patient follow-up. A reference standard was determined by surveying hospital records. No histologic biopsies were conducted. The methods of follow-up were ranked in order of preference: (1) An unmeasurable prostate-specific antigen (PSA) after radical prostatectomy (conclusion: No bone metastases); (2) a later conclusive imaging result by MRI, CT, additional ^68^Ga-PSMA PET, additional ^18^F-NaF PET, or bone scintigraphy (conclusion: Bone metastases/no bone metastases); (3) a stable PSA without treatment (Conclusion: No bone metastases); and (4) a clinical conclusion determined on the basis of a multidisciplinary team conference (conclusion: Bone metastases/no bone metastases). Follow-up method 1 was considered the best form of follow-up and was used if present. Method 4 was considered the least preferred follow-up and was considered only if none of the other 3 methods were available. Through the exclusion of patients with only method 4 available, a subgroup of patients with “most valid follow-up” was created.

### 2.6. Statistics

Descriptive statistics were conducted on the baseline clinical and epidemiological characteristics of the study population. Sensitivity, specificity, positive predictive value, negative predictive value, and accuracy of ^68^Ga-PSMA PET/CT regarding bone metastases on patient level were calculated using SPSS 25 (IBM, Armonk, NY, USA). Results are presented with 95% confidence intervals when applicable.

## 3. Results

A total of 2918 ^18^F-NaF PET/CT scans were performed in PC patients referred from the department of urology in the time period. In 51 patients (1.7%) an additional ^68^Ga-PSMA PET/CT was done since the patients had no known bone metastases and the ^18^F-NaF PET/CT was inconclusive. The characteristics of these patients are shown in Table 1. Most of the patients (39/51 = 76%) had recently been diagnosed with PC and were referred for primary staging. The rest had signs of progression of an already known PC diagnosis with eight (16%) of the patients followed with active surveillance or watchful waiting, three (6%) had biochemical recurrence after previous radical prostatectomy, and one (2%) was on androgen deprivation therapy due to lymph node metastasis at previous primary staging. Characteristics of the subset of patients referred for primary staging are also shown in Table 1. The median time between the ^18^F-NaF PET/CT and the ^68^Ga-PSMA PET/CT was 21 days (range 6–77 days).

### 3.1. Equivocal Lesions on ^18^F-NaF PET/CT

The most frequent location of equivocal lesions was the pelvis (31%), and the second most frequent site was in the ribs (27%; Table 2). Only 13% (2/16) of patients with a solitary equivocal lesion in the pelvis were according to follow-up ultimately classified as having bone metastasis. In all, 25% (13/51) of patients with equivocal lesions on ^18^F-NaF PET/CT were finally determined to have bone metastases. In patients with multiple equivocal lesions on ^18^F-NaF PET/CT, 42% (5/12) ended up with the diagnosis of bone metastases. ^18^F-NaF PET/CT would misclassify 75% (38/51) of the patients if all the inconclusive bone lesions were considered malignant.

### 3.2. Findings on ^68^Ga-PSMA PET/CT

None of the additional ^68^Ga-PSMA PET/CT scans were inconclusive regarding bone metastases. Forty-nine patients (96%) were classified correctly when ^18^F-NaF PET/CT was inconclusive. Two patients (4%) had false positive results (stable PSA without treatment for 5 and 13 months after the PET scans, respectively).

The patient-based sensitivity and specificity of the method were 100% (95% confidence interval (CI): 75%–100%) and 95% (82%–99%), respectively, for the 51 patients included in the study (Table 3). If only the 39 patients with recently diagnosed PC were considered, the sensitivity and specificity were 100% (66%–100%) and 93% (78%–99%).

The subgroup with the most valid follow-up consisted of 33 patients (Table 4). The sensitivity and specificity were 100% (95% CI: 54%–100%) and 93% (76%–99%), respectively. In this subgroup, 26 patients were referred for primary staging. The sensitivity and specificity were 100% (40%–100%) and 91% (71%–99%), respectively.

## 4. Discussion

This retrospective study has demonstrated high accuracy (96%) of ^68^Ga-PSMA PET/CT when ^18^F-NaF PET/CT is inconclusive regarding bone metastases on patient level in patients with no known bone metastasis. It is notable since inconclusive bone scans (bone scintigraphy and ^18^F-NaF PET/CT) is a well-known challenge in everyday practice. Despite the frequent challenge of inconclusive bone scans, there is currently no consensus on how to proceed with the diagnostic evaluation. The EAU-EANM-ESTRO-ESUR-SIOG guideline does not mention the management of equivocal bone scans [3]. According to National Comprehensive Cancer Network guidelines plain films, CT, MRI, F-18 sodium fluoride PET/CT or PET/MRI, C-11 choline PET/CT or PET/MRI, F-18 fluciclovine PET/CT or PET/MRI can be considered for equivocal results on initial bone scan [4].

In some studies, the imaging modalities used for follow-up have been registered. An American multicenter observational study demonstrated that 99 out of 639 (15%) bone scintigraphy scans were equivocal in men with castration-resistant PC [22]. Within 3 months, 43% of the 99 patients underwent follow-up imaging (additional bone scintigraphy, CT, MRI, and/or X-ray). No assessment of the quality of the follow-up imaging was conducted. In another study, Wondergem and colleagues observed 226 men who underwent bone scintigraphy (n=104) or ^18^F-NaF PET/CT (*n* = 122) for primary staging of PC [23] Equivocal findings warranted further diagnostic procedures in 2% of the patients in the NaF cohort and in 16% in the bone scintigraphy cohort. Follow-up imaging consisted of MRI, CT, and X-ray. The variability of follow-up imaging is consistent with other studies [24], but to our knowledge, the quality of these additional imaging procedures has not been evaluated.

Several studies have shown high diagnostic accuracy of ^68^Ga-PSMA PET/CT for bone lesions in different stages of PC [19,20,25,26,27]. Only a few studies have compared ^68^Ga-PSMA PET/CT and ^18^F-NaF PET/CT. A retrospective study examined 16 men with advanced-stage PC scheduled for radionuclide therapy [28]. ^18^F-NaF PET/CT detected 486 lesions, compared to 351 lesions detected by ^68^Ga-PSMA PET/CT. Two prospective studies, including PC patients with biochemical recurrence and patients at different stages of PC, found that both ^18^F-NaF PET/CT and ^68^Ga-PSMA PET/CT had high patient-based diagnostic performances [19,20].

**Figure 1:**
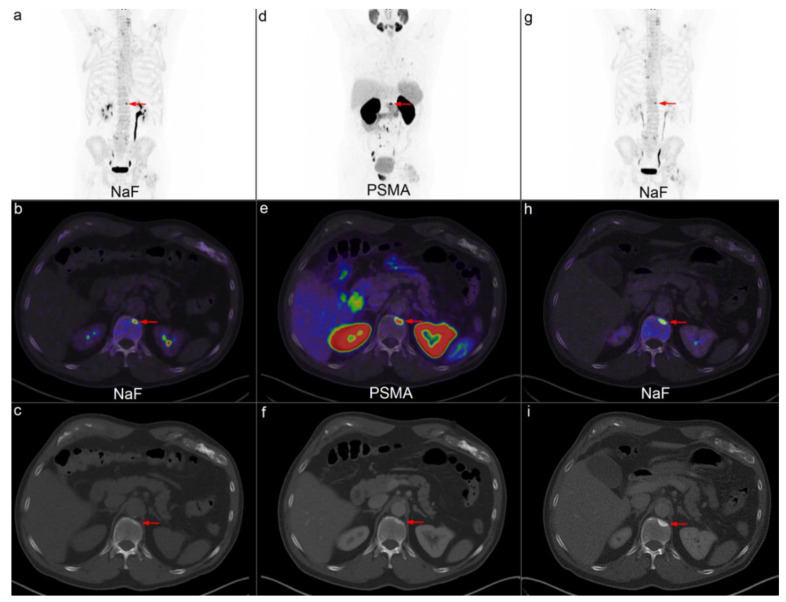
Example of a patient referred for ^18^F-NaF PET/low-dose CT (ldCT) for primary staging. An inconclusive lesion (*red arrow*) with increased uptake on ^18^F-NaF PET/ldCT is noticed in Th12 on maximum intensity projection (MIP) (**a**) and in fused axial projection (**b**). No correlating lesion is observed on ldCT (**c**). Subsequent ^68^Ga-PSMA PET/CT showed increased uptake (**d**,**e**). Nevertheless, there was no visible lesion on CT (**f**). Five months later, an additional ^18^F-NaF PET/CT scan showed increased uptake (**g**,**h**). A sclerotic lesion was visible on CT by that time (**i**). Several lymph node metastases and lung metastases are noticed on the PSMA MIP (**d**).

The benefit of additional ^68^Ga-PSMA PET/CT when ^18^F-NaF PET/CT is inconclusive could be explained by the complementary role of tracer binding in bone. Metastatic cells from PC are disseminated hematogenously to the highly vascularized bone marrow [29]. Here, the cancer cells bind to the marrow stroma cells and bone matrix. Cancer cells secrete substances that lead to bone resorption, which provides the opportunity for tumor growth. However, it is still unclear whether bone resorption precedes bone formation or vice versa [29]. ^68^Ga-PSMA binds to receptors on the tumor cells in the bone. Conversely, ^18^F-NaF is incorporated into the hydroxyapatite matrix of the bone and thereby reflects the sclerotic reaction of the bone to the tumor cells [30]. Often, a sclerotic response is visualized in subsequent morphologic imaging (Figure 1).

**Figure 2:**
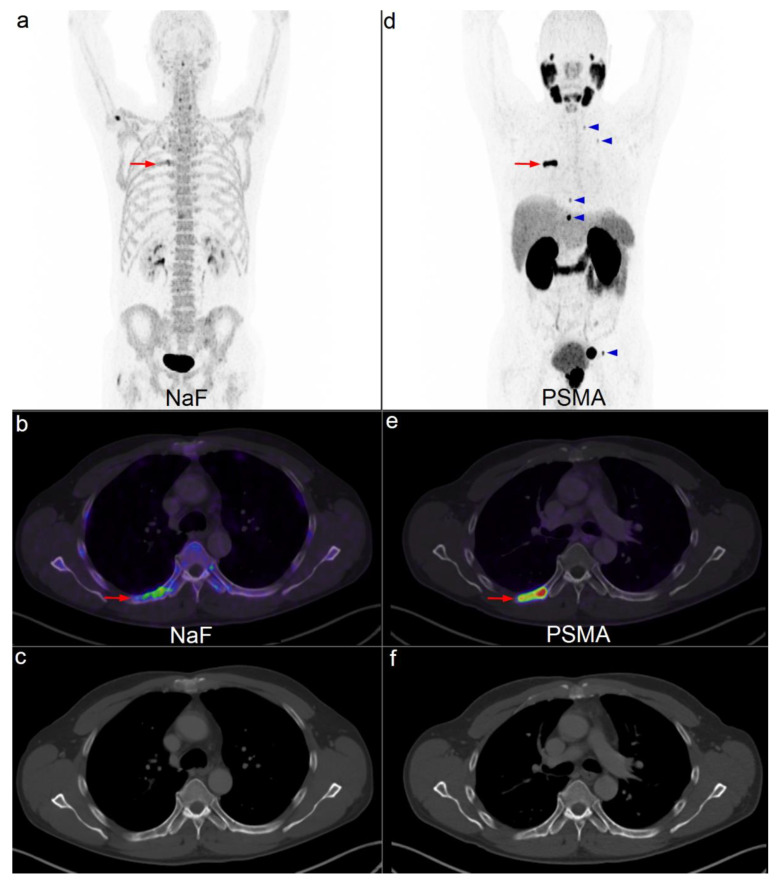
Example of a patient referred for ^18^F-NaF PET/low-dose CT for primary staging. An irregular lesion with faint uptake (*red arrow*) is visible on ^18^F-NaF PET/CT (**a**,**b**). Corresponding CT shows small, unspecific sclerotic areas (**c**). Subsequent ^68^Ga-PSMA PET/CT (**d**,**e**) shows intense uptake (*red arrow*). Several additional bone lesions with increased uptake are visible on maximum intensity projection (MIP) (**d**) (*blue arrowheads*). The corresponding CT is unchanged (**f**). PSMA MIP (**d**) also shows increased uptake in the prostate and in an enlarged lymph node in the left side of the pelvis.

Most of the inconclusive lesions on ^18^F-NaF PET/CT were located in the pelvis and ribs. Only 13% of the solitary lesions in the pelvis were ultimately classified as bone metastases. Frequently, the first bone metastases are discovered in the pelvis. Hence, it is reasonable to believe that the nuclear physician is more concerned of a lesion in the pelvis on ^18^F-NaF PET/CT. In the ribs, 21% of the solitary equivocal lesions were malignant. The high sensitivity of ^18^F-NaF PET/CT compared with bone scintigraphy allows many small lesions, often located in the ribs, to become visible. Our experience is that most lesions in the ribs are insignificant. However, sometimes bone metastasis cannot be excluded (Figure 2).

Obviously, the chosen imaging modality for detecting bone metastases depends on availability, local practice, and cost. Bone scintigraphy is widely available, relatively cheap, and the recommended imaging modality in detecting bone metastases in PC patients [3]. Some of the planar bone scintigraphies are inconclusive [8,23,31] thus potentially both misclassifies patients and/or delay treatment. The proportion of equivocal scans is markedly decreased if supplemental SPECT/CT is done and comparable to ^18^F-NaF PET/CT [8,32]. Yet, a small number of scans remain inconclusive. In this retrospective, observational study, we found that 1.7% of the 2918 ^18^F-NaF PET/CT scans in the inclusion period were equivocal and the referring physician requested additional imaging. In this setting ^68^Ga-PSMA PET/CT showed high accuracy regarding bone metastases, which was the final diagnosis in 25% of the patients. Therefore, it seems reasonable to use ^18^F-NaF PET/CT or bone scintigraphy combined with SPECT as the initial bone imaging modality and complement it with ^68^Ga-PSMA PET/CT, if available, in inconclusive cases.

In this study, no lesions were confirmed or rejected by histologic biopsy. Therefore, additional imaging, PSA tests, and decisions made at multidisciplinary team conferences were used as reference standards. Some of these reference standards were marred by high uncertainty. In these cases, the influence of ^68^Ga-PSMA PET/CT could have confounded the reference standard. However, if these cases were excluded, the data could be biased. Instead, in order to minimize the influence of ^68^Ga-PSMA PET/CT on the reference standard, a subgroup of patients with the most valid follow-up was created. In this subgroup, the high sensitivity and specificity of ^68^Ga-PSMA PET/CT were confirmed.

Another limitation of this study was its retrospective design, which may, among other things, induce selection bias. When an ^18^F-NaF PET/CT was deemed inconclusive, it was the referring urologist who decided whether the patient should be further investigated with ^68^Ga-PSMA PET/CT. No other additional imaging modalities were preferred when ^18^F-NaF PET/CT was equivocal.

In some cases, ^18^F-NaF PET/CT was conducted with low-dose CT. Even low-dose CT achieves high image quality in bone. However, the possibility cannot be ruled out that decision making might have been altered in single cases if diagnostic CT had been performed. Moreover, the low-dose CT scans were not routinely reviewed by a radiologist.

## 5. Conclusions

This study shows that ^68^Ga-PSMA PET/CT is an excellent complementary modality in PC patients without known bone metastases when ^18^F-NaF PET/CT is equivocal. Prospectively designed studies are warranted to confirm these results. Most equivocal lesions on ^18^F-NaF PET/CT were located in the pelvis and ribs. Only a minority of these lesions were malignant.

## Figures and Tables

**Table 1:** Patient characteristics.

Characteristics	All Patients *n* = 51	Patients Referred for Primary Staging *n* = 39
Age (mean and 95% CI)	67.2 (64.8–69.6)	65.6 (62.9–68.3)
**Reason for referral**		
Primary staging	39 (76%)	39 (100%)
Increasing PSA	8 (16%)	n/a
Other reasons	4 (8%)	n/a
**PSA**		
< 10 ng/ml	11 (22%)	10 (26%)
10–20 ng/ml	19 (37%)	16 (41%)
> 20 ng/ml	17 (33%)	13 (33%)
n/a ^a^	4 (8%)	0 (0%)
**Tumor stage**		
≤ T2a	22 (43%)	14 (36%)
T2b	6 (12%)	6 (15%)
≥ T2c	19 (37%)	19 (49%)
n/a ^a^	4 (8%)	0 (0%)
**Gleason**		
≤ 6	4 (8%)	2 (5%)
7 (3+4)	14 (27%)	10 (26%)
7 (4+3)	11 (22%)	10 (26%)
≥ 8	18 (35%)	17 (44%)
n/a ^a^	4 (8%)	0 (0%)
**D’Amico risk classification**		
Low risk	1 (2%)	0 (0%)
Intermediate risk	14 (27%)	12 (31%)
High risk	32 (63%)	27 (69%)
n/a ^a^	4 (8%)	0 (0%)

^a^ n/a: Not applicable. Three patients had biochemical recurrence after radical prostatectomy, and one had lymph node metastases.

**Table 2:** Anatomical locations of the equivocal lesions on ^18^F-NaF PET/CT in 51 patients.

Location of Lesion	Distribution	True Positive ^a^
Pelvis (*n* = 16)	31%	13% (2/16)
Lumbar vertebrae (*n* = 0)	0%	n/a ^b^
Thoracic vertebrae (*n* = 5)	10%	40% (2/5)
Femora (*n* = 1)	2%	0% (0/1)
Ribs (*n* = 14)	27%	21% (3/14)
Other (*n* = 3)	6%	33% (1/3)
Multiple (*n* = 12)	24%	42% (5/12)
All patients (*n* = 51)	100%	25% (13/51)

^a^ Frequency of bone metastasis in each region. ^b^ n/a: Not applicable.

**Table 3:** Patient-based diagnostic performance of ^68^Ga-PSMA PET/CT.

	All Patients *n* = 51	Patients Referred for Primary Staging *n* = 39
Sensitivity (95% CI)	100 (75–100)	100 (66–100)
Specificity (95% CI)	95 (82–99)	93 (78–99)
Positive predictive value (95% CI)	87 (63–97)	82 (54–95)
Negative predictive value	100	100
Accuracy (95% CI)	96 (87–100)	95 (83–99)

**Table 4:** Patient-based diagnostic performances of ^68^Ga-PSMA PET/CT on patients with the most valid follow-up.

	All Patients *n* = 33	Patients Referred for Primary Staging *n* = 26
Sensitivity (95% CI)	100 (54–100)	100 (40–100)
Specificity (95% CI)	93 (76–99)	91 (71–99)
Positive predictive value (95% CI)	75 (44–92)	67 (35–88)
Negative predictive value	100	100
Accuracy (95% CI)	94 (80–99)	92 (75–99)
